# Membrane Sampling Separates Naphthenic Acids from Biogenic Dissolved Organic Matter for Direct Analysis by Mass Spectrometry

**DOI:** 10.1021/acs.est.1c07359

**Published:** 2022-02-17

**Authors:** Kyle D. Duncan, Jeffrey A. Hawkes, Mykelti Berg, Bas Clarijs, Chris G. Gill, Jonas Bergquist, Ingela Lanekoff, Erik T. Krogh

**Affiliations:** † Analytical Chemistry, Department of Chemistry − BMC, Uppsala University, Husargatan 3, 751 24 Uppsala, Sweden; ‡ Department of Chemistry, Vancouver Island University, 900 Fifth Street, Nanaimo, British Columbia, Canada V9R 5S5; § Applied Environmental Research Laboratories, Department of Chemistry, Vancouver Island University, 900 Fifth Street, Nanaimo, British Columbia, Canada V9R 5S5; ∥ Department of Chemistry, University of Victoria, P.O. Box 1700, Stn CSC, Victoria, British Columbia, Canada V8W 2Y2; ⊥ Department of Environmental and Occupational Health Sciences, University of Washington, Seattle, Washington 98195, United States

**Keywords:** naphthenic acids, dissolved organic matter, membrane sampling, octanol/water partitioning, mass spectrometry, permeability, hydrophobic contaminants

## Abstract

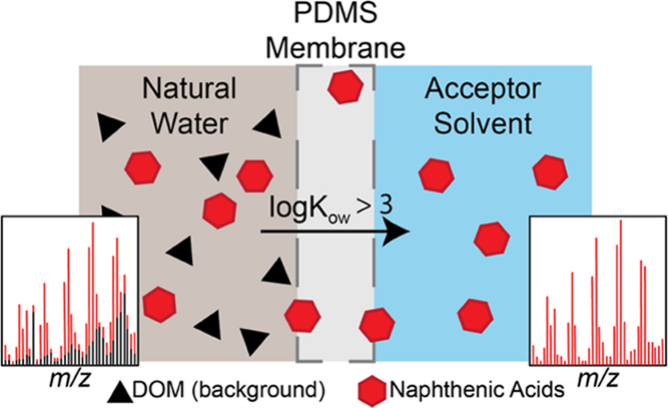

Oil sands process waters can release toxic naphthenic acids (NAs) into aquatic environments. Analytical techniques for NAs are challenged by sample complexity and interference from naturally occurring dissolved organic matter (DOM). Herein, we report the use of a poly(dimethylsiloxane) (PDMS) polymer membrane for the on-line separation of NAs from DOM and use direct infusion electrospray ionization mass spectrometry to yield meaningful qualitative and quantitative information with minimal sample cleanup. We compare the composition of membrane-permeable species from natural waters fortified with a commercial NA mixture to those derived from weak anion exchange solid-phase extraction (SPE) using high-resolution mass spectrometry. The results show that SPE retains a wide range of carboxylic acids, including biogenic DOM, while permeation through PDMS was selective for petrogenic classically defined NAs (C*_n_*H_2*n*+*z*_O_2_). A series of model compounds (log *K*_ow_ ∼1–7) were used to characterize the perm-selectivity and reveal the separation is based on hydrophobicity. This convenient sample cleanup method is selective for the O_2_ class of NAs and can be used prior to conventional analysis or as an on-line analytical strategy when coupled directly to mass spectrometry.

## Abstract 2

A simple hydrophobic membrane separates classical naphthenic acids from ubiquitous biogenic dissolved organic matter, enabling next-generation field-portable analytical methods.

## Introduction

The extraction of hydrocarbons from Canada’s oil sands deposits results in the production of enormous quantities of oil sands process-affected water (OSPW), which have been observed to exhibit both acute and chronic toxicity to a variety of organisms.^1−3^ Under the current “no release” policy, OSPW is stored on-site in large settling ponds covering in excess of 176 sq km.^4^ Consequently, there is a great deal of interest in developing viable treatment options and establishing extensive environmental monitoring campaigns. OSPWs are extraordinarily complex mixtures containing a variety of hydrocarbons, salts, suspended solids, residual bitumen, as well as water-soluble naphthenic acids (NAs).^5,6^ NAs were originally isolated from the acid extractable fraction of OSPW and described as a complex mixture of C_10_–C_30_ organic carboxylic acids represented by C*_n_*H_2*n*+*z*_O_2_, where *z* is a negative integer representing the hydrogen deficiency. While the description of naphthenic acid fraction compounds has been broadened to include a wider range of functional groups and heteroatoms,^6^ much of the toxicity has been associated with the classical “O_2_ class” of NAs.^7−10^ In particular, the monocarboxylated NAs above 17 carbons have been observed to have the highest toxicological potency.^3^ Nonetheless, there are thousands of individual NAs compounds over the mass range of 200–600 as observed by negative ion electrospray ionization mass spectrometry (ESI-MS).^11^

Monitoring NAs is important in assessing toxic pollution from OSPWs in the environment, as well as the design, optimization, and operation of engineered treatment and remediation strategies. However, the complexity of this analyte class and the structural similarity with naturally occurring dissolved organic matter (DOM) contribute significant analytical challenges for the determination of NAs in real environmental and biological samples. A great deal of research has elaborated on the structural diversity and complexity of NA fractions, which is summarized in several recent reviews.^5,12^ State-of-the-art methods for NA analysis rely on sample cleanup, high capacity chromatographic separation, and/or high-resolution mass spectrometry.^13−17^ Such methods are sensitive and can provide discrete molecular information for thousands of chemical constituents in NA mixtures. Quantitative information is also possible when appropriate analytical standards are available or the target structure is known. However, traditional chromatographic methods are labor-intensive, time-consuming, and costly, limiting their utility in applications such as process monitoring, where rapidly screening a large number of samples and/or conditions is advantageous.^15,18,19^ Direct analysis methods that obviate chromatographic separation can be prone to positive bias, particularly from naturally occurring DOM.^20−22^ It is therefore highly desirable to develop direct sampling analytical methods that exclude DOM and enable high throughput screening and simple “on-line” workflows.

At the molecular level, biogenic DOM is structurally similar to petrogenically derived NAs, as both compound classes contain carboxylic acid functional groups, alicyclic and aromatic rings, unsaturations, and heteroatoms. In fact, the distinction between DOM and NA is somewhat blurred because the definitions of these compound classes are largely operational and not based on discrete structural features. Further, both exist as complex mixtures with numerous isomeric structures. Given the ubiquity of DOM in environmental samples, often present at concentrations between 10 and 50 ppm C in inland waters,^23^ distinguishing between NAs and background DOM can be analytically challenging. This is particularly true for direct analysis methods that aim to minimize sample cleanup and chromatographic steps. Fortunately, it has been established that DOM has a wide but limited range of hydrophobic character (log *K*_ow_ 0–3.5),^24^ whereas classical NAs are significantly more hydrophobic (log *K*_ow_ 4–8).^24,25^ Therefore, classical NAs can be separated from DOM by harnessing their differential hydrophobicity and partitioning behaviors, as is done with reversed-phase chromatographic methods.

Condensed phase membrane introduction mass spectrometry (CP-MIMS) has been demonstrated as an analytical method capable of rapidly screening NAs directly in water samples, with very little or no sample preparation.^19,26−29^ In CP-MIMS, a capillary hollow fiber poly(dimethylsiloxane) (PDMS) polymer membrane is mounted on an immersion probe and connected to a flowing acceptor phase solvent. After sample acidification, protonated NAs partition into and diffuse through the PDMS into the acceptor solvent for subsequent, direct infusion and ionization in an atmospheric pressure ion source of a mass spectrometer. Suspended and ionized components (e.g., salts) do not partition into the membrane, providing an on-line sample cleanup. The technique has been employed to screen samples providing quantitative information and follow dynamic processes in the sample solution phase over time.^19,26,29−32^ While CP-MIMS has been demonstrated for the on-line analysis of trace hydrophobic analytes, there has not been a systematic evaluation to parameterize the perm-selectivity of membrane transport and the use of membrane sampling to effectively separate biogenic DOM from petrogenic naphthenic acids.

Herein, we examine the permeation of model NA compounds using CP-MIMS coupled to a triple quadrupole MS. Off-line experiments using high-resolution MS are employed to assess the molecular composition of natural waters containing both NAs and DOM, comparing PDMS membrane extracts with those from weak anion-exchange solid-phase extraction (SPE). Isocratic high-performance liquid chromatography was used to estimate log *K*_ow_ values and examine NA isomer class enrichment introduced by PDMS membrane sampling. This work aims to demonstrate a convenient and robust method for excluding DOM background and broaden NA analysis to include a wider range of MS instrumentation available in most industrial and academic labs.

## Experimental Section

### Materials, Reagents, and Complex Samples

All glassware used for DOM experiments was heated at 450 °C before use, and all solvents were ultrapure grade (>99.9%). Natural river water samples were collected from Alberta, Canada, and Uppsala, Sweden. The Canadian sample is a composite from the Athabasca River, collected in the oil sands deposit region between Fort McMurray and Fort MacKay (June 2012). The DOC concentration of this sample was measured at 19.8 ppm C prior to the experiments described here (February 2018) as nonpurgeable organic carbon using a TOC analyzer (Shimadzu TOC-L, Duisberg, Germany). The Fyrisån river sample was collected in Uppsala, Sweden, (February 2018) and typically contains 15–20 ppm DOC (not measured on this occasion). Both samples were filtered through a precombusted glass fiber filter (GF/F, 0.7 μm) and stored at 4 °C until the analysis. Stock solutions of a commercial refined mixture of naphthenic acids (Merichem Company, Houston, TX), Nordic Reservoir NOM and Suwannee River NOM (International Humic Substances Society, St. Paul, MN) were prepared gravimetrically in methanol (HPLC grade, Fisher Scientific, Ottawa, Canada). Aqueous standards were subsequently prepared in 18 MΩ-cm water (Milli-Q, Millipore, Etobicoke, Canada).

Four preparations of complex samples were considered in this study. (1) Naphthenic acids in deionized water (1 ppm Merichem), (2) Fyrisån river water (Sweden), (3) Athabasca River water (Canada), and (4) Athabasca River water with 1 ppm NAs (Merichem). These four samples were extracted by SPE and PDMS membrane as discussed below. Additionally, the NA mixture (Merichem) and the suite of model carboxylic acids was prepared in 50% acetonitrile with 20 ppb lauric acid-*d*_2_ for analysis without extraction, to provide formula lists for peak assignment.

### Preparation of Model Compounds Solutions

Model carboxylic acids with a range of octanol/water partitioning coefficients were purchased at purity >97% from Sigma-Aldrich (Oakville, Canada) and listed in Table S1. Individual stock solutions were prepared gravimetrically in HPLC grade methanol at ∼100 ppm. An ultrasonic bath (FS140, Fisher Scientific) was utilized to aid in the dissolution of the less soluble compounds with higher *K*_ow_ values. Stock solutions were subsequently diluted in methanol for direct infusion MS experiments or deionized water for CP-MIMS experiments. All aqueous solutions used in CP-MIMS experiments contained ≤0.1% methanol cosolvent. One drop of 6 M HCl was added to 40 mL of aqueous solutions immediately before making CP-MIMS measurements to adjust the pH to ∼3, converting carboxylates to their protonated (neutral) form for membrane sampling.

### CP-MIMS Triple Quadrupole Experiments for Investigating the Permeation of Model Compounds

Experiments were conducted with an in-house constructed immersion “J-probe” with a 2 cm length of PDMS capillary hollow fiber membrane (Silastic tubing, Dow Corning, Midland; OD = 0.64 mm; ID = 0.30 mm; 170 μm thickness) mounted on 22 gauge stainless steel hypodermic tubing.^26,30^ A mobile methanol acceptor phase containing 5 ppb of a lauric acid-*d*_2_ internal standard passed through the membrane lumen at 75 μL/min using a syringe pump (Harvard Apparatus Pump 11 Elite, St. Laurent, Canada) and was continuously infused to the electrospray ionization mass spectrometer. Aqueous samples were analyzed by immersing the probe into a 40 mL sample vial fitted with a septum cap cut to hold the J-probe assembly,^26^ mixed at 1000 rpm using a magnetic stir bar and plate. Each model compound was measured in triplicate at four concentrations. Direct injection of a single high concentration standard was performed in triplicate at the end of each run to account for changes in day-to-day instrument sensitivity.

Samples were analyzed by an electrospray ionization triple quadrupole mass spectrometer (Micromass Quattro Ultima LC, Waters Micromass, Altrincham, U.K.). Negative ion ESI was used with a capillary voltage of −3.2 kV and entrance cone voltage of 30 V. A dwell time of 0.25 s was used for each *m*/*z* monitored. A desolvation gas of ultrahigh-purity nitrogen (Praxair, Nanaimo, Canada) was operated at 750 L/h and maintained at a temperature of 225 °C. Multiple reaction monitoring (MRM) was used to detect model compounds (Table S2). Compounds with no observable product ions were measured at low collision energy (2 eV), with the precursor *m*/*z* selected for detection. Data was processed using the instrument operating software (MassLynx Ver 4.1, Waters Micromass). All experiments were performed under ambient temperature and pressure conditions (∼20 °C, ∼101 kPa).

Direct infusion QqQ-MS analysis was carried out to establish the linear dynamic range for each model compound and to quantify concentrations in the methanol acceptor phase for CP-MIMS experiments. To accomplish this, a 6-port valve fitted with a 200 μL injection loop (PEEK tubing ID = 0.076 cm) was utilized to produce 2.7 min signals for replicate injections separated by a methanol baseline. A methanol solution of lauric acid-*d*_2_ (10 ppb) was analyzed before and after each model compound to account for any signal drift during continuous operation. For each injection, at least 1 min (∼400 data points) of steady-state signal intensity was averaged and used in the subsequent data analysis. Details of the workflow in determining the risetime (τ), the steady-state concentration of model compounds in the acceptor phase ([*X*_i_^SS^]_MeOH_), and the conditional partition constant (*K*′_PDMS_) are presented in the Supporting Information.

### Octanol/Water Coefficients of Model Compounds Solutions

The *K*_ow_ values for model compounds were experimentally determined using the well-established correlation between log *K*_ow_ and the HPLC retention times for a structurally related series of compounds.^24,33^ Calculated *K*_ow_ values for the protonated form of syringic, 4-phenylbutyric, and pyrenebutyric acids were employed as a training set to predict the values for the remaining compounds using isocratic reversed-phase chromatography (details below) on C_18_ resin in an acidified mobile phase using eq 1, where *t*_r_ is retention time (Table S1).
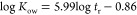
1

### Off-Line Membrane Extraction for HRMS Analysis of NA and DOM Samples

Off-line membrane extractions were performed with a 40 cm length of the same capillary hollow fiber PDMS membrane (above) submerged in 30 mL of a magnetically stirred aqueous sample adjusted to pH < 4 with HCl to facilitate carboxylic acid transport. Permeating molecules were dissolved in a methanol acceptor solvent within the lumen of the membrane. After a 30 min equilibration, the methanol acceptor solvent was flushed for 30 min using a syringe pump at 50 μL min^–1^ and collected for subsequent analysis by direct infusion HRMS. Following each sampling event, the membrane was washed with clean methanol for at least 30 min.

### Solid-Phase Extraction of Complex Samples

Weak anion exchange solid-phase extraction cartridges (SPE–WAX, 200 mg, Phenomenex, Torrance, CA) were conditioned with 5 mL of MeOH followed by 5 mL of deionized water. A 10 mL aqueous sample was adjusted to a pH of 7.0 by adding 1% formic acid and 1% ammonium hydroxide dropwise. The sample was passed over the SPE–WAX cartridge by gravity. The cartridge was then washed with 25 mM ammonium acetate (2 mL) and MeOH (1 mL) before being dried under N_2_. The organic material was eluted from the cartridge with 5% ammonium hydroxide in methanol (2 mL), which was flushed with a 20 mL syringe of air after gravity drainage. Extracts were dried under N_2_ and then re-dissolved in acetonitrile (0.5 mL) with an overall concentration factor of 20. Before the analysis, an aliquot (0.25 mL) was mixed with 0.1% ammonium hydroxide in methanol (0.25 mL), and 80 μL of this sample was spiked with 20 μL of a 100 ppb stock of lauric acid-*d*_2_ (analytical concentration: 20 ppb). The samples were introduced to the HRMS Orbitrap (described below) with a syringe pump operating at 5 μL/min.

### High-Resolution Mass Spectrometry (HRMS)

Complex samples were analyzed by Orbitrap Velos Pro mass analyzer (Thermo Fisher, San Jose, CA) using negative mode electrospray ionization (ESI) at spray voltage 3.1 kV. Data were acquired using Thermo Fisher Scientific Xcalibur software (version 3.0) with a mass range of 100–1000 *m*/*z* and an instrumental resolution setting of 100 000. The instrument was externally calibrated with the manufacturer’s calibration mix spiked with l-arginine (*m*/*z* 173.104) and hydrocinnamic acid (*m*/*z* 149.061) to improve the mass accuracy at lower masses. Briefly, 1 × 10^6^ ions were accumulated in each transient in the Orbitrap, and 150 transients were averaged for each sample. Mass lists were internally calibrated using *m*/*z* 255.23321 and 283.26425, two common laboratory contaminants present in all samples, and were exported along with peak intensities to Microsoft Excel.

### High-Performance Liquid Chromatography (HPLC)

HPLC was conducted with an Agilent 1100 system fitted with a binary pump and well plate autosampler. Samples were prepared in 100% MeOH and were injected at 1 μL onto a C18 column (Waters Atlantis T3; 3 μm 2.1 × 150 mm^2^), and analytes were separated using an isocratic method (80% methanol, 20% water, 0.1% formic acid, 250 μL min^–1^ flow rate). The flow was split so that about 98% of eluent was directed to waste, and ∼5 μL min^–1^ was diverted to the ESI-HRMS. Orbitrap MS data were collected as before and were internally calibrated using *m*/*z* 255.23321 and 283.26425 with Thermo ReCal off-line software. The resulting Thermo Fisher.raw files were converted to .mzXML format using ReAdW software.

### Data Processing for Exact Mass and Molecular Formulae

Sample mzXML data (HPLC-MS) and xlsx files (direct infusion MS) were processed to assign formulas to signals in MATLAB (Version 2017b) as in previous studies,^34,35^ with further details found in the Supporting Information. Formulas containing C, H, or O, plus lauric acid-*d*_2_ were allowed as for assignment, with H/C ratio of 0.3–2.0 and O/C ratio 0–1 and the requirements that formulas be valence neutral and mass 100–700. A total of 64 334 potential formulas were allowed, leading to 2003 assigned formulas that were within 1.5 ppm of the calculated exact mass.

## Results and Discussion

### NAs and DOM are Structurally Similar but Chemically Diverse

**Figure 1:**
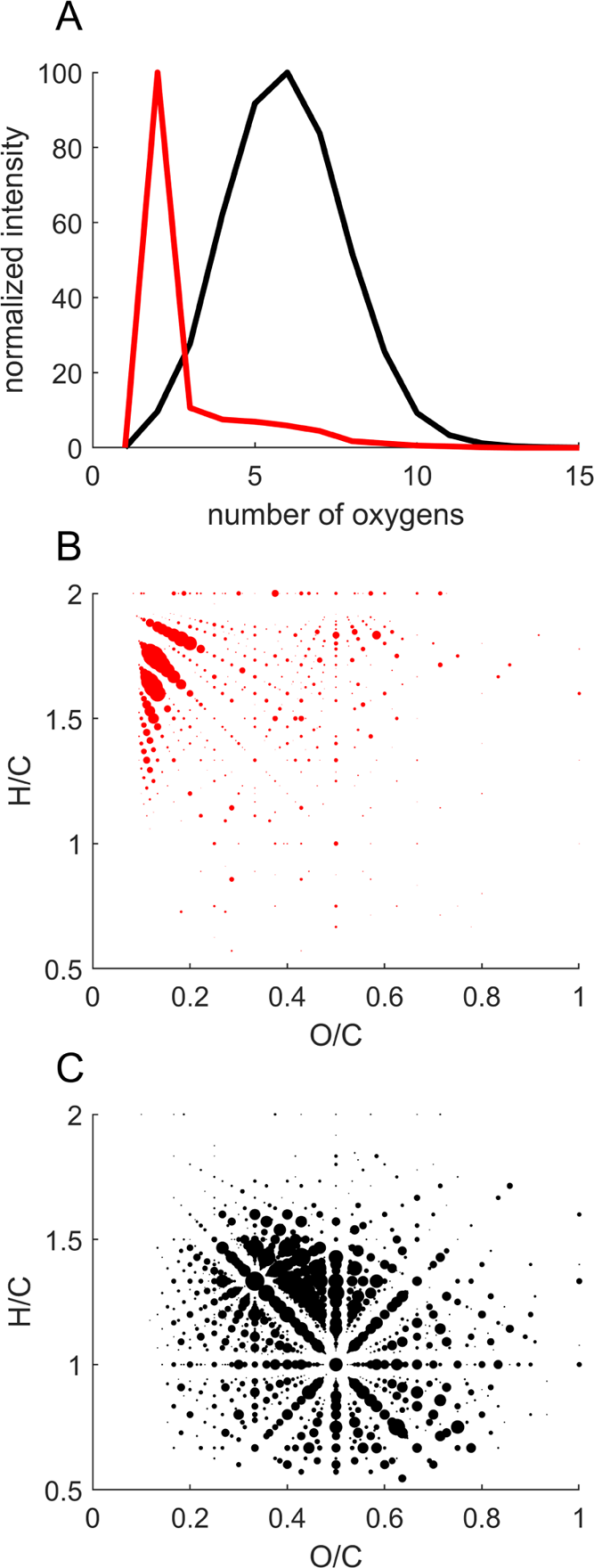
Comparison of direct infusion (−)ESI-HRMS data for Merichem NA mixture (red, 1 ppm) and Nordic Reservoir NOM (black, 50 ppm). (A) Oxygen distribution (weighted intensity). (B) Van Krevelen diagram for Merichem NA mixture. (C) Van Krevelen diagram for Nordic Reservoir NOM; point size corresponds to peak intensity relative to lauric acid-*d*_2_ internal standard.

One approach to identify classical NAs in complex aqueous samples containing biogenic DOM is to leverage the high resolving power of mass spectrometric methods and generate accurate masses for molecular formula assignment. Figure 1 displays HRMS data for the direct infusion of commercial Merichem NA and Nordic Reservoir NOM standard solutions at a mass resolution of ∼100 000. The ESI(−) HRMS mass spectra for [M – H]^−^ ions show that considerable overlap between NAs and DOM will occur at nominal mass resolution (Figure S1). However, after molecular formula assignment, clear compositional differences emerge in terms of oxygen content and hydrophobic character. Figure 1A depicts the oxygen atom distribution, revealing that this NA mixture was comprised of compounds with relatively low oxygen content, heavily dominated by the O_2_ compound class. In contrast, the DOM sample displays a broader distribution of oxygenated species with a higher oxygen content (e.g., O_5_ to O_12_). These trends are visualized in Van Krevelen diagrams, where compounds with high H/C and low O/C ratios are associated with a greater hydrophobic character, as shown in red for the authentic NA standard (Figure 1B). Conversely, compounds in biogenic DOM are more hydrophilic, with a wider range of O/C ratios and lower H/C ratios (Figure 1C). While the molecular diversity in both NAs and DOM mixtures will vary between sources and can be different from commercially available standard mixtures, it is generally recognized that DOM is more oxygenated and consequently more hydrophilic than classically defined NAs regardless of their source. For example, we observe similar characteristics for the DOM in the Fyrisån River (Figure S2) consistent with reports for other DOM sources.^24,34,36^ It is worth noting that OSPW contains a broader range of naphthenic acid fraction compounds and may not be well represented by Merichem NA mixtures. However, it does contain significant quantities of the classically defined NAs, which are associated with the aquatic toxicity of OSPW. Nonetheless, given that the molecular formulae assignments distinguishing NAs from biogenic DOM rely on the accurate mass obtained from HRMS, commonly available and affordable low-resolution mass spectrometers, such as triple quadrupole or ion trap instruments, cannot be used to easily analyze NAs in the presence of DOM without some form of molecular separation (e.g., chromatography or membrane permeation).

### PDMS Perm-Selectivity of NA Model Compounds is Based on Hydrophobicity

**Figure 2:**
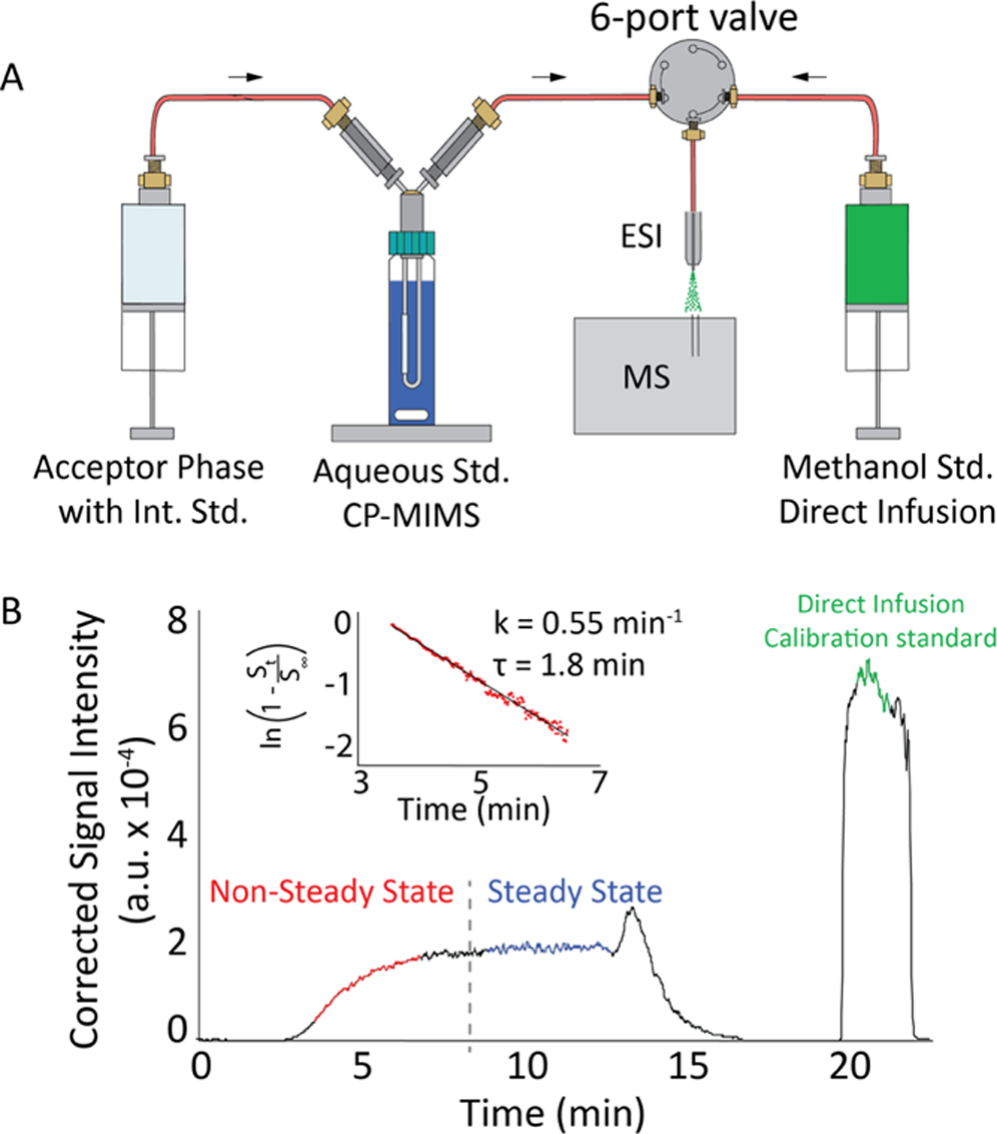
Membrane permeation studies of model compounds for the determination of *K*′_PDMS_ showing a schematic of the experimental setup for direct infusion and CP-MIMS (panel A) and typical time-resolved MS signal (panel B). The time series shows the signal response to a stepwise increase in aqueous phase concentration of a model compound at *t* = 3 min. The nonsteady state signal (red) is fit to yield the natural risetime from the slope of the line (inset). *S_t_* represents the signal at time *t*, *S*_∞_ is the signal at steady state, *k* is the pseudo-first-order time constant for diffusion through the PDMS membrane, and the natural risetime τ is 1/*k*. At *t* = 13 min; the membrane was washed in methanol and at 20 min, the 6-port valve was switched, and a standard solution was directly infused.

To characterize the selective permeability of PDMS membrane sampling, a series of 10 model carboxylic acids with octanol/water partition coefficients spanning over 5 orders of magnitude were investigated by CP-MIMS coupled to a triple quadrupole mass spectrometer (Table 1). Figure 2 illustrates the experimental apparatus used, with the ability to directly infuse methanolic standards or the membrane permeate acceptor phase to the ESI source of a mass spectrometer with a 6-port valve.

**Table 1:** Experimentally Determined Physicochemical Properties of Model Compounds

name	log *K*_ow_	log *K*′_PDMS_
syringic acid	1.06 (0.77)^a^	na
6-hydroxy-2-naphthoic acid	1.36	–3.36
phenylacetic acid	1.48	–2.16
4-phenylbutyric acid	2.08 (2.47)^a^	–2.42
2-naphthoic acid	2.26	–2.26
6-phenylhexanoic acid	2.98	–1.71
cyclohexanebutyric acid	3.70	–0.97
pyrenebutyric Acid	4.89 (4.79)^a^	–0.19
dihydroabietic acid	6.87	0.95

a *K*_ow_ values used in the training set to determine others (training set values in brackets).

Figure 2B shows a representative time series data set. At 3 min, the CP-MIMS immersion probe is placed into a well-stirred aqueous standard solution containing model NA compounds. The nonsteady state rise in the mass spectrometer signal is due to the diffusion-controlled permeation of analyte through the PDMS membrane. The data between roughly 3–7 min corresponding to 20–90% of the signal rise (red) is log-transformed to yield a linear fit used to calculate the natural risetime (τ), which is inversely proportional to the diffusivity (*D*). The average steady-state signal between 9 and 12 min is proportional to the analyte concentration. At 20 min, the 6-port valve was actuated to directly infuse a methanolic standard solution of the model compound and used to calibrate the concentration in the acceptor phase. The permeation efficiency of each model compound was thus determined by measuring its concentration in the methanol permeate (acceptor phase) relative to its concentration in the corresponding prepared aqueous standard solution (donor phase).

Membrane permeation is governed by the product of partitioning (*K*_PDMS_) and the diffusivity (*D*) through the polymer.^37,38^ The diffusivity is largely governed by the hydrodynamic volume of the permeant and is inversely proportional to the signal risetime (τ). We therefore define a conditional partition constant, *K*′_PDMS_, for each model compound as follows, where [*X*_i_] and τ_i_ represent the concentration and risetime of model compound i, respectively.
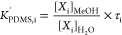
2While the values of *K*′_PDMS_ are specific to the conditions of the CP-MIMS experiment (membrane geometry, acceptor phase flow rates, etc.), they can be used as a relative measure of PDMS perm-selectivity when comparing compounds under identical conditions. We have included additional background on permeation and the data analysis in the Supporting Information.

**Figure 3:**
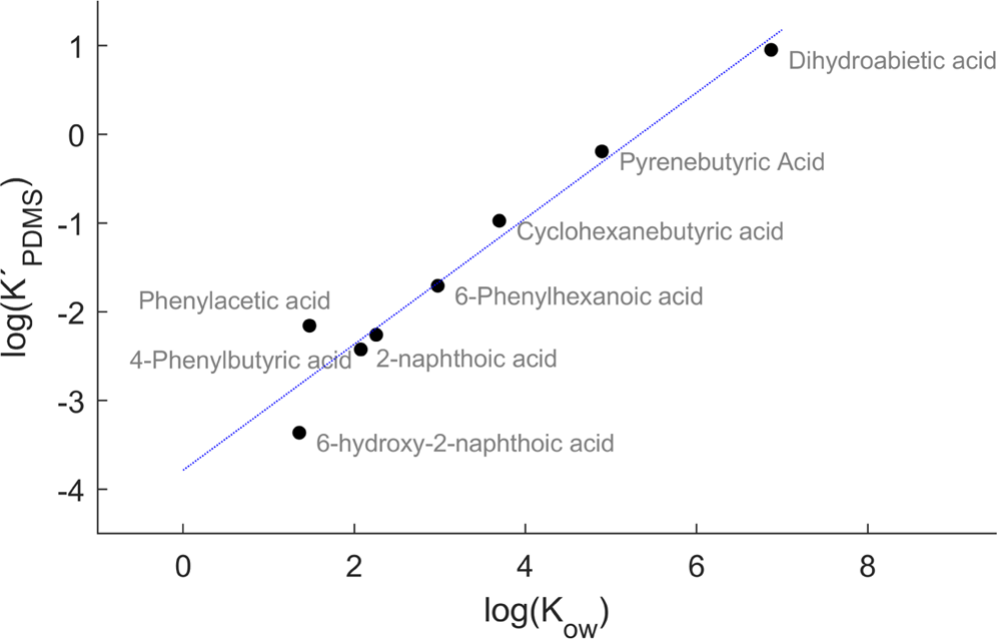
Measured log *K*′_PDMS_ of the model compounds across the PDMS membrane versus the estimated log *K*_ow_ from HPLC retention times. Syringic acid (log *K*_ow_ ∼1) was not detected by membrane sampling even at aqueous concentrations >5 ppm and therefore is not included here.

Plotting the experimentally derived log *K*′_PDMS_ values obtained from CP-MIMS permeation experiments against the log *K*_ow_ (Table 1), we observe a linear free energy relationship (Figure 3).

This confirms that the observed perm-selectivity of PDMS in the CP-MIMS experiments is driven by the intrinsic hydrophobic character of the permeant, as reported by the octanol/water partition coefficient. Equation 3 is critical in parameterizing membrane transport, an important contributor to both the analytical sensitivity and selectivity of CP-MIMS.
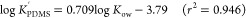
3Hydrophilic molecules partition less into the PDMS, resulting in a reduced concentration gradient across the membrane. Hence, even low molecular weight (i.e., small hydrodynamic volume) compounds will suffer a low permeation efficiency if they are hydrophilic, resulting in reduced CP-MIMS analytical sensitivity. Furthermore, the quantitative structure–activity relationship established here suggests that the CP-MIMS technique could be used to predict octanol/water partition coefficients for structurally related series of compounds in complex mixtures and at low analyte concentrations.

### PDMS Membrane Sampling is Selective for Naphthenic Acids

**Figure 4:**
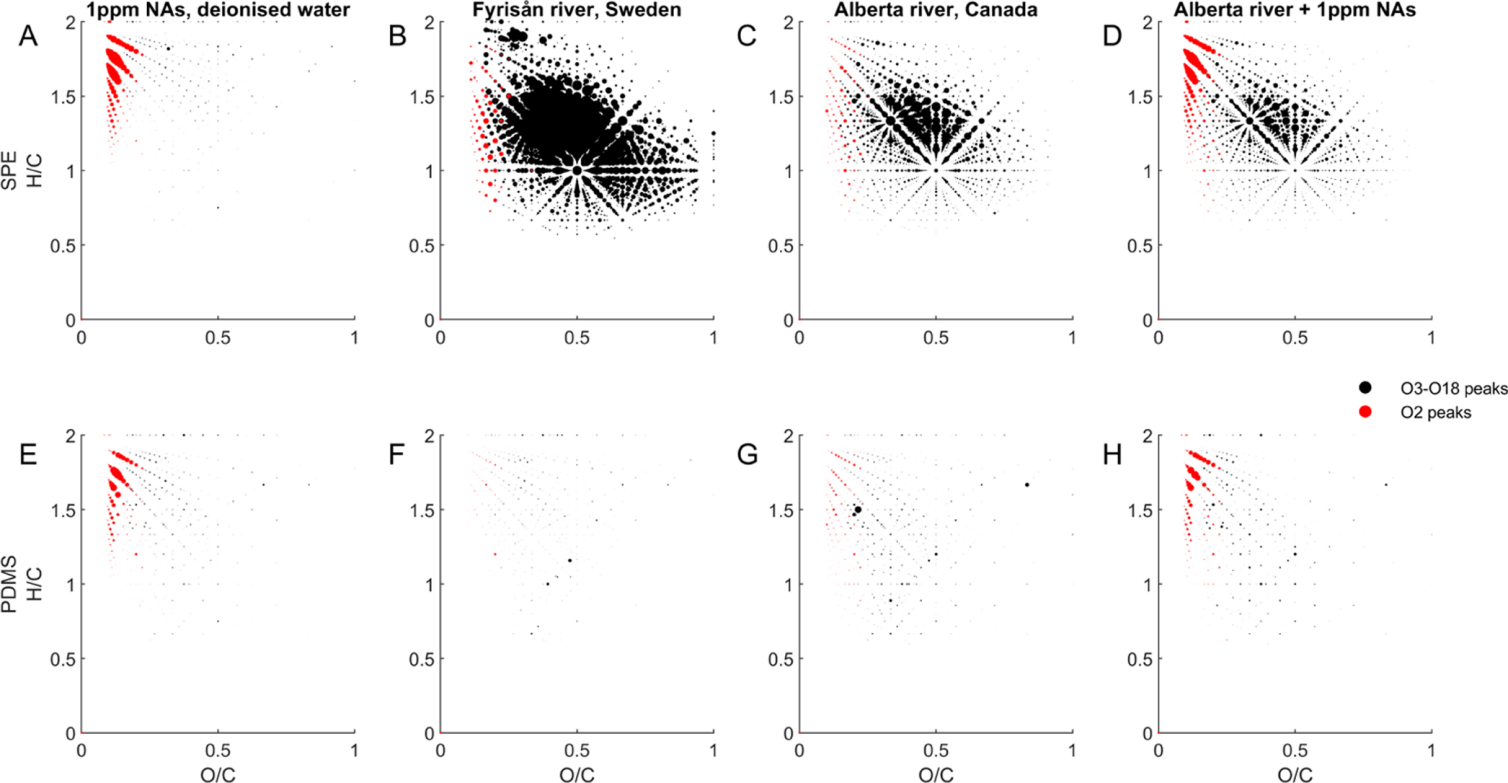
Van Krevelen diagrams showing extracted molecular masses of trace organic compounds in water samples using SPE extraction (top; A–D) and PDMS membrane sampling (bottom; E–H). Point size indicates the intensity relative to the lauric acid-*d*_2_ internal standard. Panels (A, E) are deionized water fortified with 1 ppm NA standard, (B, F) are native river water from Uppsala, Sweden, panels (C, G) are composite samples from Alberta, Canada, and panels (D, H) are the same composite river samples fortified with 1 ppm NA standard. The preconcentration factor for the SPE extractions was 20×.

To demonstrate the selectivity of membrane sampling for NAs over DOM, two natural river water samples were investigated, one from the oil sands region of Alberta (Canada) and the other in Uppsala (Sweden), with no heavy oil deposits or local NA sources. All samples were analyzed by direct infusion HRMS (Orbitrap), and molecular formulae calculated from the accurate masses were used to generate Van Krevelen diagrams. In Figure 4, we compare sample cleanup by SPE–WAX^20^ to the membrane permeate. Panels A–D present four distinct water samples after SPE–WAX of samples are adjusted to pH 7, whereas panels E–H are the PDMS membrane extracts of the same water samples after adjusting to pH ∼3. Data points are intensity-scaled to the internal standard lauric acid-*d*_2_, and those with molecular formulae meeting the criteria for classical NAs (C*_n_*H_2*n*+*Z*_O_2_) are depicted in red. To visualize trends over the wide range of signal intensities, a variation of these plots is found in Figure S3, where the point sizes have been scaled to the square root of the normalized signal intensity.

The Van Krevelen plots of a NA standard mixture in deionized water illustrate that NAs are present in both the SPE–WAX and PDMS extracts (panels A and E, respectively). On the other hand, the river Fyrisån (Sweden) containing ca. 15 ppm C show that the SPE–WAX method retains biogenic DOM, whereas the membrane is essentially impermeable to this class of compounds (panels B and F, respectively). This led to the extremely low total intensity of detected compounds in the membrane extraction, as shown in panel F, consistent with the absence of NAs in this sample. A composite Athabasca river sample from northern Alberta (Canada) shows a high DOM background by SPE–WAX (panel C), which is absent in the PDMS membrane extract (panel G). This is consistent with the measured dissolved organic carbon of ca. 20 ppm. After fortification with 1 ppm of a commercial NA mixture, the SPE–WAX method detects NAs as well as the DOM compounds (panel D). In contrast, the PDMS membrane extract is clearly selective for NAs in the presence of DOM (panel H). Similar experiments using Suwannee River NOM again demonstrate PDMS membrane selectivity for NAs (Figure S4). Interestingly, the Van Krevelen plots for panels C and G in Figure 4 suggest that low concentrations of O_2_ NAs are present in the Athabasca composite. It should be noted, however, that not all hydrophobic DOM is necessarily NAs,^39^ nor are all NAs anthropogenic.^40^ While the perm-selectivity of PDMS favors the hydrophobic O_2_ NA class over biogenic DOM under acidic conditions, the broader class of NAFCs, including more polar heteroatom constituents, are not membrane-permeable under the current conditions.^30^ Nonetheless, it does provide a convenient diagnostic technique to monitor the presence of O_2_ NAs, which are a recognized contributor to OSPW aquatic toxicity. Overall, these data indicate that NA and DOM are both extracted with SPE–WAX, while PDMS membrane sampling is selective for classical NAs and supports our previous findings, where DOM had negligible effects on NA quantification with membrane sampling.^19,29,31^

While solid-phase extraction offers a robust method for NA preconcentration and the removal of background salts that complicate MS analyses, our data indicates that SPE based on anion affinity will also retain biogenic DOM. Ultimately, this creates a necessity for high-resolution mass spectrometry (accurate formula assignment) to discriminate between trace NAs in natural waters containing biogenic DOM. On the other hand, PDMS membrane sampling was shown to be highly selective for classical NAs in the presence of naturally occurring DOM. This is consistent with our earlier observations that the membrane is perm-selective for the O_2_ class of NAs^30^ and more recent control experiments showing no positive bias in the quantitation of classical NAs in waters impacted by diluted bitumen in the presence of high DOM loads up to 50 ppm C.^29^ The selectivity of O_2_ NAs and exclusion of DOM are significant findings, due to recent studies identifying the O_2_ class of NAs as some of the most highly toxic components in the acid extractable organic fractions of oil sands process waters.^7^ Furthermore, it suggests that PDMS membrane sampling may be used for the determination of classical NAs in environmental samples, even using low-resolution mass spectrometry or nonspecific techniques, such as NMR or IR.^7,12,22^ This approach would enable more cost-effective and flexible methods for sample collection, cleanup, and detection of NA pollution in remote areas, including via portable mass spectrometry.

### HPLC–HRMS of Natural Waters and NA Mixtures

**Figure 5:**
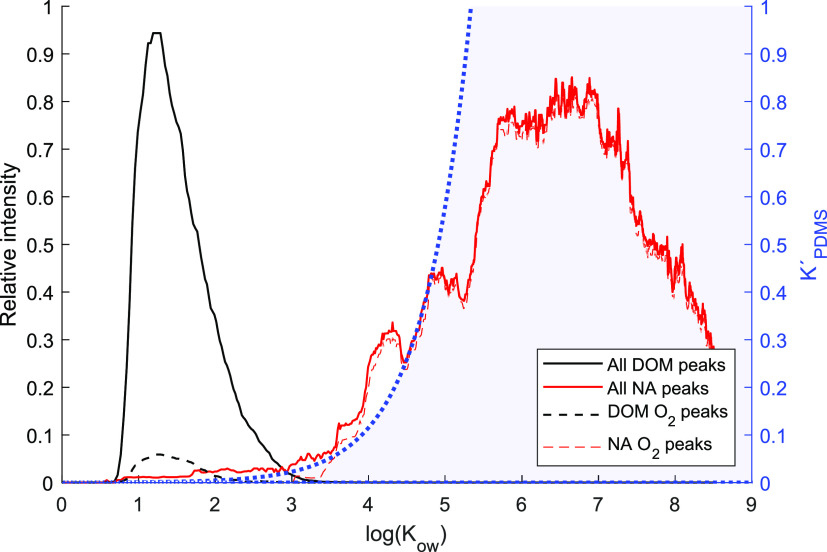
HPLC-MS of Merichem NA (red) and the SPE extract of Fyrisån river water (Sweden; black). The chromatograms represent the sum of all peaks detected (solid lines), and O_2_ formulas detected in both samples (dotted lines, ×10 for DOM). The chromatograms are smoothed to 10-point medians. The DOM peaks generally fall in the log *K*_ow_ <3 range, whereas peaks in the Merichem sample generally have a log *K*_ow_ >3. The O_2_ formulas follow the same ranges as the bulk organic matter in each case. The dotted blue line is derived from eq 3 for *K*′_PDMS_ and represents the relative mass transport efficiency of membrane sampling as determined from the permeation behavior of a series of model compounds.

We analyzed the solid-phase extract of the river Fyrisån (Sweden) and the Merichem mixture of NAs using the same isocratic HPLC-HRMS technique as the model compounds. The resulting separation of the two complex mixtures based on hydrophobicity is clear (Figure 5). Using the retention times of model compounds and/or isomer classes identified from the HRMS data and eq 1, we estimate the corresponding log *K*_ow_ values displayed on the *x*-axis. All DOM-related compounds (black) elute early with a corresponding range of log *K*_ow_ values between about 0.8 and 3, consistent with previous observations.^24^ Conversely, the NA-related compounds (red) elute much later, corresponding to higher log *K*_ow_ values ranging from roughly 4 to 8. This distinct separation between the more hydrophilic DOM and more hydrophobic NAs falls between log *K*_ow_ values of 3 and 4. This corresponds to the point at which membrane permeation becomes more favorable, as depicted by the exponential increase in *K*′_PDMS_ (dotted blue line) superimposed on Figure 5, derived from eq 3. Interestingly, the O_2_ compounds found in the Fyrisån river DOM (“O_2_ peaks”) had lower retention times than the O_2_ peaks in the NA mixture, indicating that the O_2_ peaks in DOM from this river are not naphthenic acids and may be misidentified as such by other techniques.^39^ The results presented here reveal that at environmental concentrations, DOM is effectively excluded by membrane sampling, whereas more hydrophobic NAs undergo selective membrane transport through PDMS.

### Effect of Membrane Sampling on NA Isomer Class Distributions

We have previously noted that the isomer class distribution of O_2_ class NA mixtures observed by CP-MIMS^26,30,41^ is remarkably similar to those published by others.^5^ The hydrophobicity of classical NAs typically ranges over log *K*_ow_ 4–8 and increases with carbon number and degree of hydrogen saturation.^25^ This can be seen in the HRMS data set for the Merichem NA mixture (Figure S3) and suggests that higher molecular weight NA components may be enriched by membrane sampling relative to lower molecular weight NAs. However, the greater permeability of larger NA components is offset to some extent by their lower diffusivity (*D*), as reported by their slower natural risetimes (τ). Panel B in Figure S5 compares the relative ion abundances between direct infusion, SPE, and PDMS. Both the SPE and PDMS treatments appear to under-represent the smaller NAs (*m*/*z* < 223) relative to direct infusion without sample preparation, whereas PDMS and direct infusion treatments appear to converge for higher molecular weight isomer classes (*m*/*z* > 237). Given that all sample cleanup procedures impart some bias when applied across a diverse mixture of molecules, the effects observed here are relatively modest, with membrane samplings imparting minimal bias among O_2_ isomer classes. However, it would be interesting to explore the extent to which membrane sampling affects the distribution of individual NAs further in complex mixtures, including OSPW extracts. It should be noted that many of the constituents in the broader class of NA fraction compounds,^6^ which include heteroatoms (N, S) and additional O, are sufficiently polar or remain ionized in solution and therefore not sampled by membrane extraction. While other hydrophobic compounds, such as polycyclic aromatic hydrocarbons, are known to permeate the PDMS membrane,^42^ they do not bias the results presented here, as they are not ionized by ESI(−). On the other hand, some nonpetrogenic carboxylic acids (i.e., resin acids from the pulp and paper industry) may impart a positive bias, if present.

The examination of complex aqueous samples at high mass resolution clearly demonstrates differences in the chemical composition and membrane permeability of DOM and NAs. We demonstrate that appreciable membrane transport is associated with compounds with log *K*_ow_ >4, while compounds with log *K*_ow_ <3 remain largely in the aqueous phase. This effectively separates classical NAs (O_2_) from DOM in environmental samples and is considerably more selective than weak anion exchange SPE. Consequently, on-line membrane extraction provides a convenient sample introduction platform for the analysis of the toxic O_2_ NAs in natural waters when coupled to mass spectrometry for rapid screening and high throughput analysis. The results further suggest that membrane extraction may be an effective sampling or sample cleanup strategy for hydrophobic contaminants when coupled with conventional off-line analytical methods or more portable (low resolution) instruments with the aim of simplifying NA analysis for on-site and industrial applications.

## Supporting Information Available

Background permeation theory; model compound structures and *K*_ow_; low-resolution mass spectrometry settings for model compounds; membrane permeation parameters for model compounds; high-resolution full scan mass spectra for Nordic Reservoir NOM and Merichem naphthenic acids; comparison of direct infusion (−)ESI-HRMS data for Merichem NAs and Fyrisån River DOM; rescaled Van Krevelen diagrams for complex mixtures; Van Krevelen diagrams for Suwannee River NOM membrane extractions; and comparison of relative extractions of NAs for SPE and PDMS (PDF)
