# Supplementary material for: Membrane Sampling Separates Naphthenic Acids from Biogenic Dissolved Organic Matter for Direct Analysis by Mass Spectrometry
Source: Environ Sci Technol. 2022 Feb 17;56(5):3096–105. doi: 10.1021/acs.est.1c07359 (PMC8892831; doi:10.1021/acs.est.1c07359)
Supplement: Supplementary file 1 — es1c07359_si_001.pdf [file es1c07359_si_001.pdf]

Supplemental Information

Membrane sampling separates naphthenic acids from biogenic dissolved organic matter for direct analysis by mass spectrometry

Authors:
Kyle D. Duncan*,¥,[1,2], Jeffrey A. Hawkes*,[1], Mykelti Berg[3], Bas Clarijs[1], Chris G. Gill[3,4,5], Jonas Bergquist[1], Ingela Lanekoff[1], and Erik T. Krogh¥,[3,4]

Affiliations:
1. Analytical Chemistry, Department of Chemistry - BMC, Uppsala University, Husargatan 3, 751 24, Uppsala, Sweden
2. Department of Chemistry, Vancouver Island University, 900 Fifth Street, Nanaimo, BC, Canada, V9R 5S5
3. Applied Environmental Research Laboratories, Department of Chemistry, Vancouver Island University, 900 Fifth Street, Nanaimo, BC, Canada, V9R 5S5
4. Department of Chemistry, University of Victoria, PO Box 1700, Stn CSC, Victoria, B.C., Canada
5. Department of Environmental and Occupational Health Sciences, University of Washington, Seattle, Washington, 98195, United States

* K.D. and J.H. contributed equally to this manuscript

¥ Corresponding authors:
Kyle.Duncan@viu.ca
Erik.Krogh@viu.ca

The SI contains a total of 11 pages, including Background Theory, 3 Tables, 6 Figures, and References.

Table of Contents:

Background Permeation Theory……………………………………………………………………………S3-S4

Table S1………S5

Table S2………S6

Table S3………S6

Figure S1……S7

Figure S2……S8

Figure S3……S9

Figure S4……S10

Figure S5……S11

References………………………………………………………………………………………………………S11

Background Permeation Theory

Membrane permeability is governed by Fick's Law and is proportional to the product of the partitioning coefficient (K) of a permeant into the membrane and its diffusivity (D) through the membrane media.[1,2]

$$P \propto K\,D \qquad \text{Equation S1}$$

Operationally, we measure the steady-state concentration ratio of the permeant in the acceptor phase (methanol) to that in the donor phase (water) and combine this with the time dependent signal of the permeant as it reaches steady-state. In a typical CP-MIMS experiment, the MS signal rises in response to a step increase in concentration of a permeant in the donor phase as a concentration gradient is established across the membrane. The signal eventually reaches a steady-state value that is proportional to the concentration of the permeant in the donor phase. The time required to reach steady-state is inversely proportional to the diffusivity (D) of the permeant in the membrane material.

$$D \propto \frac{1}{\tau} \qquad \text{Equation S2}$$

The natural risetime (τ) is determined by fitting the time-dependent non-steady state signal to first order kinetics. We therefore define a relative conditional partition constant based on the CP-MIMS experiments using a PDMS membrane as follows.

$$K'_{pdms,i} = \left(\frac{[X_i]_{MeOH}}{[X_i]_{H2O}}\right)\tau_i \qquad \text{Equation S3}$$

Data Work up and Calculation of K'_{PDMS} for model compounds

A series of methanol stock solutions (*n*=4) were directly infused in triplicate to the mass spectrometer under conditions identical to those used in the CP-MIMS experiments to determine the linear dynamic range for each model compound. All subsequent CP-MIMS experiments preformed with aqueous standards were carried out within the established linear dynamic range. Signals were corrected for instrument drift using a continuously infused lauric acid-d_2 internal standard in the acceptor phase. Time resolved mass spectrometry signals (S_t) were smoothed using a 5-pt boxcar and normalized to the internal standard signal to yield a corrected signal (S'_t) as follows, where $S(IS)_t$ is the time dependent signal from the internal standard.

$$S'_t = \frac{S_t}{S(IS)_t} \qquad \text{Equation S4}$$

After immersing the membrane probe into an aqueous standard, the mass spectrometry signal rises in response to the step increase in concentration in the donor solution. The steady-state concentration of each compound in the methanol acceptor phase then determined by averaging *ca.*60 seconds of steady-state signal, background subtracting, and using the slope from a 1-pt

calibration obtained immediately after the permeation experiment using Equation S5, where $[X_i^{SS}]_{MeOH}$ is the steady-state concertation of compound (*i*) in the methanol acceptor phase, SS_i' is the corrected steady state mass spectrometer signal, S_i^{DI} is the average signal intensity resulting from triplicate direct infusions of a methanol standard, and $[X_i^{DI}]_{MeOH}$ is the known concentration of compound *i* in the methanol standard within the linear dynamic range.

$$[X_i^{SS}]_{MeOH} = \frac{SS_i'}{S_i^{DI} \Big/ [X_i^{DI}]_{MeOH}} \qquad \text{Equation S5}$$

The concentration of each model compound in the methanol acceptor phase was determined using a single methanol standard injected in triplicate (*m*=3) immediately after the end of each membrane permeation experiment to account for intra-day variability. The non-steady state signal was used to determine a characteristic risetime for each model compound using the natural log transformed (base *e*) time dependent signal over 20 - 90% of max signal intensity, where S_0 is the background signal and SS is the signal at steady-state.

$$ln\left(1 - \frac{S_t' - S_0}{SS - S_0}\right) \qquad \text{Equation S6}$$

A plot of this function versus time yields a straight line with a slope = $1/\tau$. All experiments were performed in triplicate and visually inspected for anomalous behaviour. Some runs were discarded due to excessive pump noise or ESI spray instability leading to poor linear fits. The characteristic risetime (τ) was then calculated in minutes. Combining this with the calculated concentration of each compound in the acceptor phase (Equation S5), the known aqueous concentration (donor phase), we report the relative conditional partition constants ($K'_{pdms,\,i}$).

Table S1. Model Compound Structures and Octanol-Water Partition Coefficients

Compound	CAS	Structure	Log K_{ow} (calc*)	Log K_{ow} (exp**)
Syringic acid	530-57-4	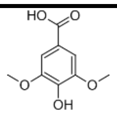	0.77	1.07
6-Hydroxy-2-naphthoic acid	16712-64-4	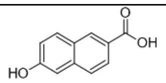		1.35
Phenylacetic acid	103-82-2	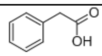		1.46
4-Phenylbutyric acid	1821-12-1	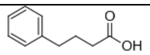	2.47	2.07
2-Naphthoic acid	93-09-4	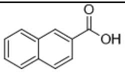		2.26
6-Phenylhexanoic acid	5581-75-9	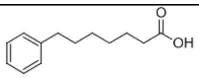		2.96
Cyclohexanebutyric acid	4441-53-8	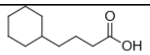		3.69
Pyrenebutyric acid	3443-45-6	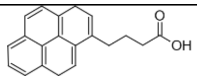	4.79	4.90
Dihydroabietic acid	19402-28-9	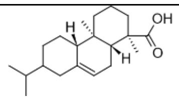		6.93

*Calculated log K_{ow} values were obtained for three model compounds in their protonated form using ChemAxon and used as an HPLC retention time training set.

**Experimental log K_{ow} values were obtained from HPLC retention times (Equation 1) using a reverse phase isocratic method (80% methanol, 20% water, 0.1% formic acid, 250 µl min^{-1} flow rate) on a C18 column (Waters Atlantis T3; 3 µm 2.1x150mm) on an Agilent 1100 with a binary pump. Identical conditions were used to estimate the log K_{ow} values for the remaining compounds and mixtures of Fyrisån DOM and Merichem NAs[2].

Table S2. Mass Spectrometer Parameters for CP-MIMS of Model Compounds

Compound	Cone Voltage (V)	Precursor *m/z*	Collision Energy	Product *m/z*
**Syringic acid**	-20	197.0	20	120.9
**Phenylacetic acid**	-15	135.0	9	91.9
**6-hydroxy-2-naphthoic acid**	-25	186.9	16	143.9
**4-Phenylbutyric acid**	-25	163.2	18	91.0
**6-Phenylhexanoic acid**	-30	191.1	2	191.1
**2-naphthoic acid**	-20	170.9	15	126.9
**Cyclohexanebutyric acid**	-30	169.0	2	169.0
**Pyrenebutyric Acid**	-30	288.3	17	216.3
**Dihydroabietic acid**	-30	303.3	2	303.3
**Lauric acid d_2**	-28	201.1	2	201.1

Membrane permeation was measured by CP-MIMS using a triple quadrupole mass spectrometer (Micromass Quattro Ultima) with negative ion ESI (capillary voltage of -3.2 kV, entrance cone voltage of 30 V). A dwell time of 0.25 s was used for each MRM transition.

Table S3. Perm-selectivity Parameters of Model Compounds

Compound	Concentration ratio (10^{-3})[a]	τ (mins)[b]	K'_{pdms}(10^{-3})[c]
**Syringic acid**	NA	NA	NA
**6-Hydroxy-2-naphthoic acid**	0.0680	6.38	0.434
**Phenyl acetic acid**	1.14	6.11	6.98
**4-Phenylbutyric acid**	2.46	1.53	3.76
**2-Naphthoic acid**	3.59	1.53	5.51
**6-Phenylhexanoic acid**	15.3	1.28	19.6
**Cyclohexanebutyric acid**	72.0	1.47	106
**Pyrene butyric acid**	89.6	7.17	642
**Dihydroabietic acid**	602	14.90	8970

[a] Steady-state concentration in flowing methanol accepter phase relative to concentration in aqueous standard solution (donor phase).
[b] Natural risetime from log transformed non-steady signal between 20-90% of steady state intensity.
[c] Conditional partition constants in PDMS under conditions of CP-MIMS experiment.

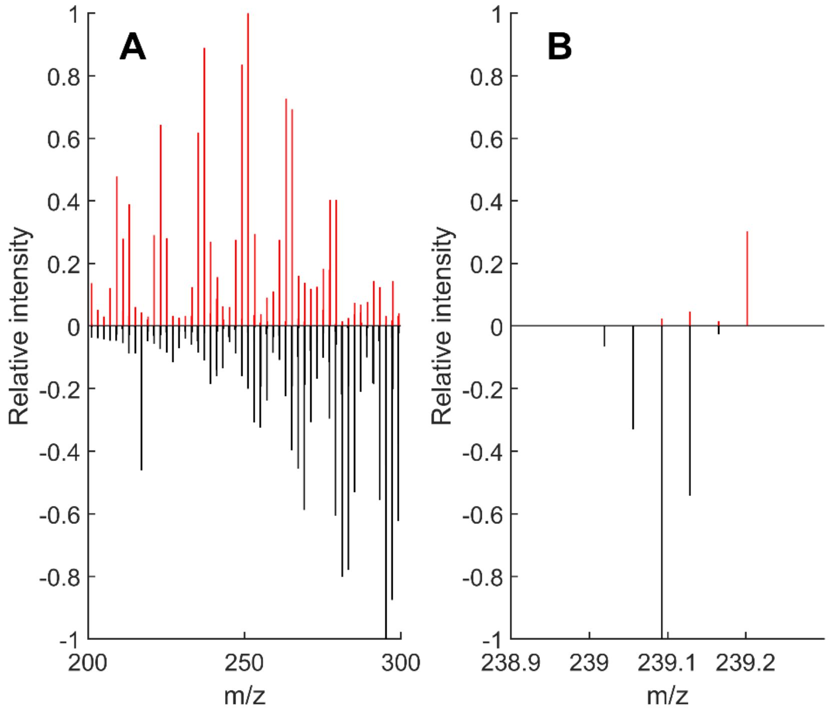

Figure S1: Full scan mass spectra for Merichem NA (top, red) and Nordic Reservoir (bottom, black) complex mixtures. Panel A shows between *m/z* 200 and 300 by direct infusion HRMS, and panel B represents peaks found in a typical nominal mass (*m/z* 239).

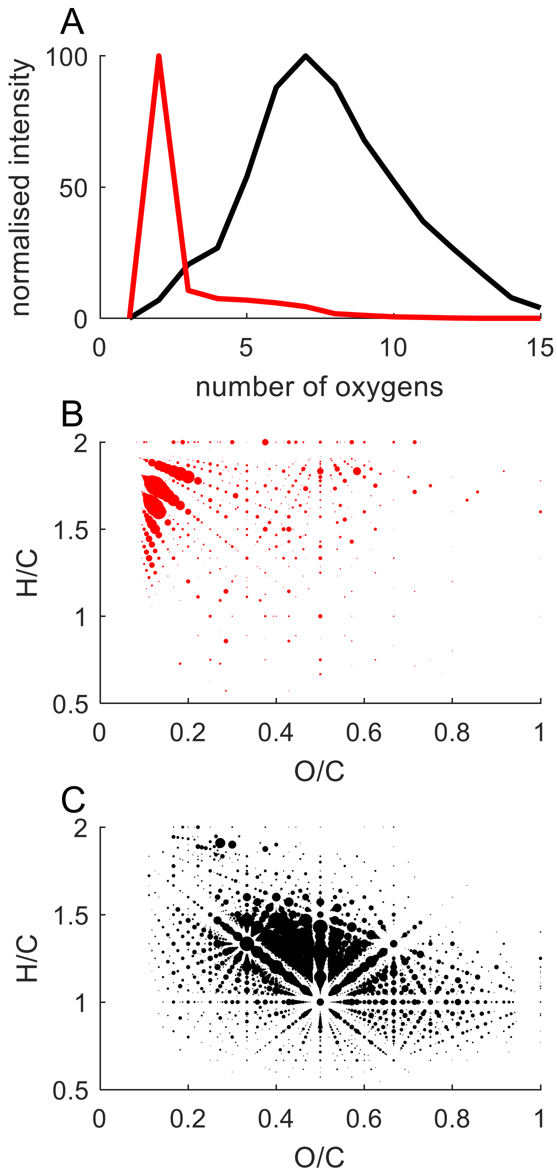

Figure S2: Comparison of direct infusion (-)ESI - HRMS data for Merichem NA mixture (red, 1 ppm) and Fyrisån River DOM (black, 50 ppm). A: Oxygen distribution (weighted intensity). B: Van Krevelen diagram for Merichem NA mixture. C: Van Krevelen diagram for Fyrisån River DOM; point size corresponds to peak intensity relative to lauric acid-d_2 internal standard.

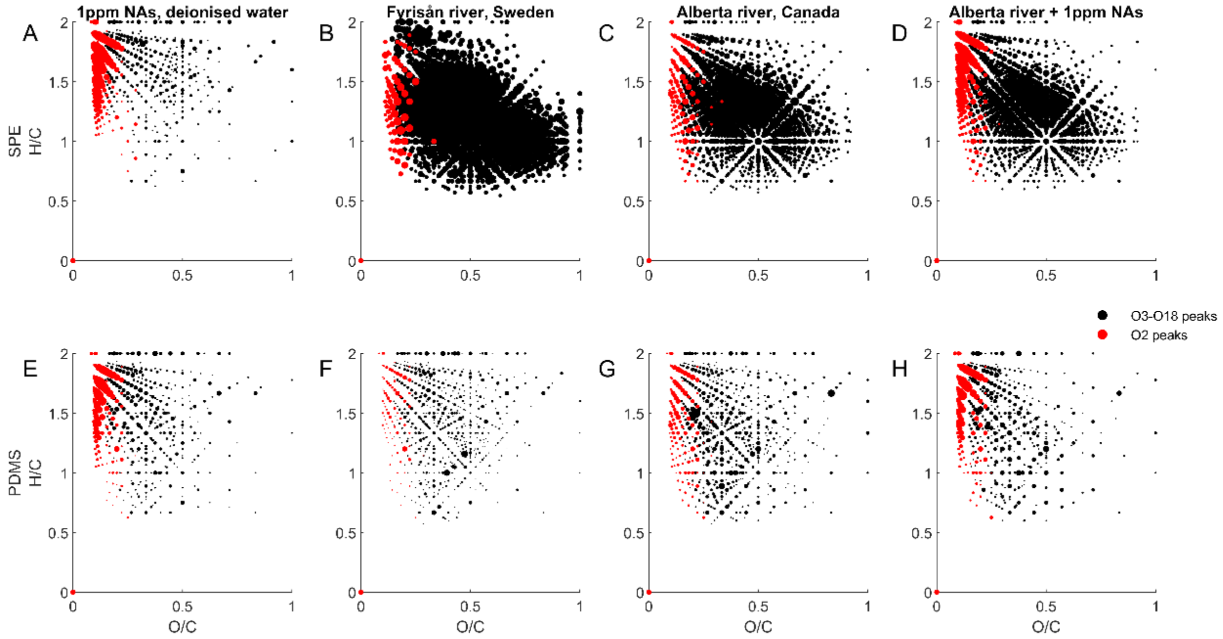

Figure S3: Van Krevelen diagrams showing the same data as in Figure 4 re-scaled to visualize low intensity peaks. Point size was scaled by the square root of the intensity relative to lauric acid-d_2 internal standard multiplied by 10. Extracted molecular masses of trace organic compounds in water samples using SPE extraction (top; A-D) and PDMS membrane sampling (bottom; E-H). Panel A/E is deionized water fortified with 1 ppm NA standard, B/F is native river water from Uppsala, Sweden, panel C/G is a composite sample from Alberta, Canada, and panel D/H is same composite river sample fortified with 1 ppm NA standard. The preconcentration factor for the SPE extractions was 20x.

$$Point\ size = \sqrt{\frac{Peak\ intensity}{Lauric\ acid\ intensity} \times 10}$$

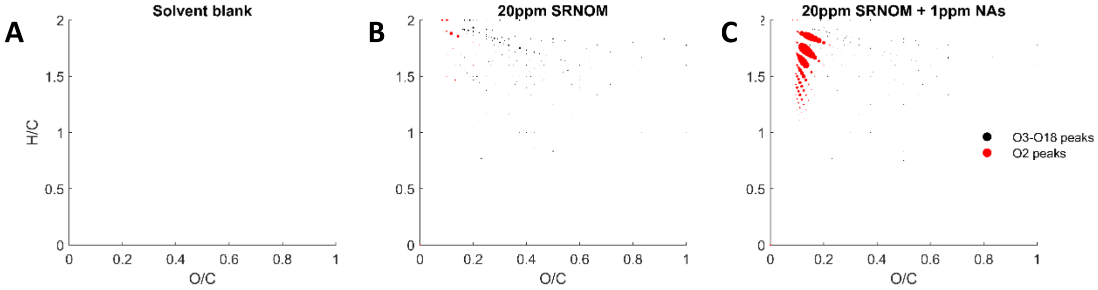

Figure S4: Van Krevelen diagrams for membrane extractions of DI water (A), 20 ppm Suwannee River NOM, and 20 ppm Suwannee River NOM spiked with 1 ppm Merichem naphthenic acids. Aqueous samples were extracted as described in the Experimental using a capillary hollow fibre PDMS polymer membrane. Suwannee river NOM was purchased from the International Humic Substances Society. Data was collected on an Orbitrap Exploris 120 in the negative ion mode between *m/z* 100 and 1000. The sample was introduced at 5 µL per minute with an inlet temperature of 325 ˚C and a capillary voltage of -3500 V. The S-Lens RF level was set to 70, and the maximum ion time was set to 0.1 ms.

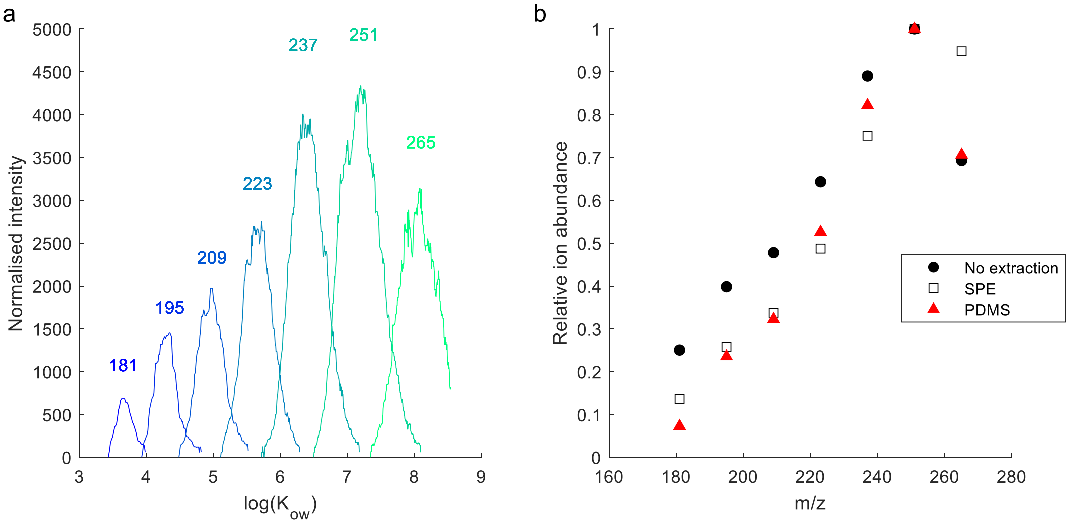

Figure S5: a) Extracted ion chromatograms of a series of NAs ($C_nH_{2n-4}O_2$) from HPLC-MS analysis of the Merichem reference mixture expressed in terms of calculated log K_{ow}, with the nominal mass of the NA indicated above the trace. b) The relative abundance of the same series of acids by direct infusion without extraction and after SPE and PDMS extraction. The two extraction methods are biased towards the higher mass NAs, which are more hydrophobic.

References:

(1) LaPack, M. A.; Tou, J. C.; Enke, C. G. Membrane Mass Spectrometry for the Direct Trace Analysis of Volatile Organic Compounds in Air and Water. *Anal. Chem.* **1990**, *62* (13), 1265–1271. https://doi.org/10.1021/ac00212a013.

(2) Janes, D. W.; Durning, C. J.; van Pel, D. M.; Lynch, M. S.; Gill, C. G.; Krogh, E. T. Modeling Analyte Permeation in Cylindrical Hollow Fiber Membrane Introduction Mass Spectrometry. *J. Memb. Sci.* **2008**, *325* (1), 81–91. https://doi.org/10.1016/j.memsci.2008.07.033.
